# Impact of the Breakpoint Region on the Leukemogenic Potential and the TKI Responsiveness of Atypical *BCR-ABL1* Transcripts

**DOI:** 10.3389/fphar.2021.669469

**Published:** 2021-06-30

**Authors:** Michele Massimino, Elena Tirrò, Stefania Stella, Livia Manzella, Maria Stella Pennisi, Chiara Romano, Silvia Rita Vitale, Adriana Puma, Cristina Tomarchio, Sandra Di Gregorio, Agostino Antolino, Francesco Di Raimondo, Paolo Vigneri

**Affiliations:** ^1^ Department of Clinical and Experimental Medicine, University of Catania, Catania, Italy; ^2^ Center of Experimental Oncology and Hematology, A.O.U. Policlinico “G. Rodolico - S. Marco”, Catania, Italy; ^3^ Department of Transfusional Medicine, Maria Paternò-Arezzo Hospital, Ragusa, Italy; ^4^ Division of Hematology and Bone Marrow Transplant, A.O.U. Policlinico “G. Rodolico - S. Marco”, Catania, Italy; ^5^ Department of Surgery, Medical and Surgical Specialities, University of Catania, Catania, Italy

**Keywords:** CML, BCR-ABL1 fusion transcripts, TKIs, nilotinib, DC2, SH3

## Abstract

Chronic Myeloid Leukemia (CML) is a hematological disorder characterized by the clonal expansion of a hematopoietic stem cell carrying the Philadelphia chromosome that juxtaposes the *BCR* and *ABL1* genes. The ensuing *BCR-ABL1* chimeric oncogene is characterized by a breakpoint region that generally involves exons 1, 13 or 14 in *BCR* and exon 2 in *ABL1*. Additional breakpoint regions, generating uncommon *BCR-ABL1* fusion transcripts, have been detected in various CML patients. However, to date, the impact of these infrequent transcripts on *BCR-ABL1*-dependent leukemogenesis and sensitivity to tyrosine kinase inhibitors (TKIs) remain unclear. We analyzed the transforming potential and TKIs responsiveness of three atypical *BCR-ABL1* fusions identified in CML patients, and of two additional *BCR-ABL1* constructs with lab-engineered breakpoints. We observed that modifications in the DC2 domain of BCR and SH3 region of ABL1 affect BCR-ABL1 catalytic efficiency and leukemogenic ability. Moreover, employing immortalized cell lines and primary CD34-positive progenitors, we demonstrate that these modifications lead to reduced BCR-ABL1 sensitivity to imatinib, dasatinib and ponatinib but not nilotinib. We conclude that BCR-ABL1 oncoproteins displaying uncommon breakpoints involving the DC2 and SH3 domains are successfully inhibited by nilotinib treatment.

## Introduction

The BCR-ABL1 oncoprotein is the molecular hallmark of chronic myeloid leukemia (CML) and transforms the hematopoietic stem cell by modulating several intracellular mediators involved in survival and proliferation (Cilloni and Saglio, 2012; Ishii et al., 2015; Manzella et al., 2016). *BCR-ABL1* is a fusion gene derived from the t (9; 22) reciprocal translocation that joins part of the *BCR* (Breakpoint Cluster Region on chromosome 22) and of the *ABL1* (Abelson murine leukemia viral oncogene homolog 1 on chromosome 9) messenger RNAs. The regions generally involved in this rearrangement are exons 1, 13 or 14 of *BCR* (e) and exon 2 of *ABL1* (a). Fusions involving one of the aforementioned exons give rise to the more common *BCR-ABL1* transcripts e1a2 (expressed in ∼20% of acute lymphoblastic leukemia), and e13a2 or e14a2 (generally detected in CML) (Laurent et al., 2001). These common *BCR-ABL1* isoforms can be expressed at different times - or simultaneously - in the same patient during the course of the disease (Gong et al., 2017; Massimino et al., 2019a; Stella et al., 2019b). However, published data demonstrates that the t (9:22) reciprocal translocation can involve additional *BCR* and *ABL1* exons, thus generating several uncommon *BCR-ABL1* fusion transcripts such as e6a2, e8a2, e19a2, e1a3, e13a3 or e14a3 (Burmeister and Reinhardt, 2008; Gong et al., 2017). The ensuing variations in the BCR and ABL1 functional domains encompassing the breakpoint region affect the oncoproteins structural organization and may influence its leukemogenic potential. Published data show that *BCR-ABL1* fusion transcripts characterized by a shorter BCR contribution - i.e., lacking both the DH-GEF and PH domains (*BCR-ABL1* ^e1a2^ and *BCR-ABL1* ^e1a3^) or only the DH-GEF region (*BCR-ABL1* ^e6a2^ and *BCR-ABL1* ^e8a2^) - trigger different signaling networks despite a kinase activity similar to that of conventional (e1a2, e13a2 and e14a2) *BCR-ABL1* isoforms (Reckel et al., 2017). Furthermore, the response to the tyrosine kinase inhibitor (TKI) imatinib depends - at least in part - on the size of the DC2 domain as individuals expressing e13a2 *BCR-ABL1* (i.e., with a shorter DC2 region) display an inferior outcome compared to those with e14a2 (which comprises a longer DC2 domain) (Jain et al., 2016; Ercaliskan and Eskazan, 2018). It should also be noted that the SH3 and SH2 regions of *ABL1* (encoded by exons 2 and 3), interact intramolecularly with the ABL1 kinase domain, thus playing a critical role in BCR-ABL1-mediated cell survival and leukemogenesis as well as TKI responsiveness (Sherbenou et al., 2010; Grebien et al., 2011).

Although different reports have described the clinical history of CML patients displaying uncommon *BCR-ABL1* transcripts (Gui et al., 2015; Ha et al., 2016; Cai et al., 2018; Chisti and Sanders, 2018; Qin et al., 2018), to date, the specific contribution of each different BCR and ABL1 domains modified in infrequent *BCR-ABL1* fusions remain unclear. We therefore analyzed the effect of uncommon *BCR-ABL1* breakpoints on the oncoprotein’s kinase efficiency, signal transduction, leukemogeneic potential and TKI sensitivity. To this end, we generated five different *BCR-ABL1* transcripts: three previously identified in CML patients (*BCR-ABL1* ^INS/Del^, *BCR-ABL1* ^e13a3^ and *BCR-ABL1* ^e14a3^), and two (*BCR-ABL1* ^∆DC2^ and *BCR-ABL1* ^∆SH3^) artificially engineered to investigate the role of the DC2 (in BCR) and SH3 (in ABL1) domains. Using *in-vitro* and *ex-vivo* models, we demonstrate that modifications in the DC2 and SH3 domains affect BCR-ABL1 signal transduction, leukemogenic potential and TKI response.

## Methods

### Identification of Atypical *BCR-ABL1* Transcripts

Total RNA extracted from primary CML cells was subjected to RT-PCR using the indicated primers as previously described (Massimino et al., 2019b): Fw BCR-10: 5′-TAT​GAC​TGC​AAA​TGG​TAC​ATT​CC-3′ and Rv ABL1-4: 5′-TCG​TAG​TTG​GGG​GAC​ACA​CC-3′. Atypical breakpoint regions corresponding to *BCR-ABL1* ^INS/Del^, *BCR-ABL1* ^e13a3^ and *BCR-ABL1* ^e14a3^ fusions were confirmed by Sanger sequencing. For *BCR-ABL1* ^INS/Del^, cDNA sequencing identified the lack of exons 13 and 14 of *BCR* and the partial deletion of exon 2 in *ABL1* with a 39 bp insert that matched a genomic region from 29534693 to 29534732 (GRCh38) corresponding to chromosome 20. The insertion/deletion produced a breakpoint between exon 12 in *BCR* and exon 2 in *ABL1*, generating the *BCR-ABL1* ^e12a2^ transcript that we refer to as *BCR-ABL1* ^INS/Del^.

### Cell Lines and Drugs

Ba/F3 (mouse pro-B cells) cell lines were purchased by DSMZ (German Collection of microorganisms and Cell Cultures GmbH) and cultivated in RPMI (Sigma) plus 10% WEHI3B conditioned medium. Rat1 cells (a gift of J.Y.J. Wang, University of California, San Diego School of Medicine, United States) were cultivated in D-MEM high glucose (Sigma). All growth media were supplemented with 10% fetal bovine serum (FBS) (Euroclone), 2 mM glutamine, 50 µg/ml streptomycin and 100 µg/ml penicillin (All from Sigma). For drug treatments, imatinib (IM) and nilotinib (NIL) were provided by Novartis, dasatinib (DAS) by Bristol Myers Squibb while ponatinib (PON) was purchased by Selleckchem.

### Isolation and Expansion of CD34-Positive Progenitors

Immunomagnetic separation of bone marrow leukemic CD34-positive progenitors expressing the e14a3 *BCR-ABL1* fusion was performed as previously described (Massimino et al., 2014). CD34-positive cells derived from healthy donors were obtained from ALLCELLS. CD34-positive cells were maintained in Stem Span SFEM supplemented with cytokines at low concentrations (Flt-3 ligand: 5 ng/ml, stem cell factor: 5 ng/ml, interleukin 3: 1 ng/ml, interleukin 6: 1 ng/ml) (all from Stem Cell Technologies).

### Generation of Lentiviral Vectors

The pLEX empty vector (EV) (Dharmacon) was used as a backbone to obtain all lentiviral vectors described below.

FLAG- and 6xHIS-*BCR-ABL1* ^WT(e14a2)^ were obtained as previously reported (Massimino et al., 2014) and used as a template to generate all *BCR-ABL1* deletion mutants.

To obtain the pLEX-FLAG-*BCR-ABL1* ^∆DC2^ and pLEX-FLAG-*BCR-ABL1* ^∆SH3^ constructs, the *BCR* and *ABL1* sequences were separately amplified using the primers reported in Table 1.

**TABLE 1:** BCR and ABL1 primer sequences to generate ΔDC2 and ΔSH3 FLAG-BCR-ABL1 deletion constructs.

		pLEX-FLAG-*BCR-ABL1* ^∆DC2^	pLEX-FLAG-*BCR-ABL1* ^∆SH3^
BCR portion	Fw	5′-ACT​AGT​GCC​ACC​ATG​GAT​TAC​AAG​GAT​GAC​GAC​GAT​AAG​ATG​GTG​GAC​CCG​GTG​GGC-3′	5′-ACT​AGT​GCC​ACC​ATG​GAT​TAC​AAG​GAT​GAC​GAC​GAT​AAG​ATG​GTG​GAC​CCG​GTG​GGC-3′
		Rv	5′-CGG​CCG​CCT​CTG​AAA​CAC​TTC​TTC​TG-3′	5′-CGG​CCG​CCT​TCA​CTG​GGT​CCA​GCG​AGA​A-3′
ABL1 portion	Fw	5′-GCG​CCG​CGA​AGC​CCT​TCA​GCG​GCC​AG-3′	5′-GCG​GCC​GCC​TGG​AGA​AAC​ACT​CCT​GGT​AC-3′
		Rv	5′-ACG​CGT​CTA​CCT​CTG​CAC​TAT​GTC​ACT-3′	5′-ACG​CGT​CTA​CCT​CTG​CAC​TAT​GTC​ACT-3′

All *BCR* portions were cloned in the pLEX-EV using Spe-I and Not-I restriction sites and each construct was subsequently subjected to ligation with his respective *ABL1* counterpart using the Not-I and Mlu-I enzymes.

To generate the pLEX-FLAG-*BCR-ABL1* ^INS/Del^, pLEX-FLAG-*BCR-ABL1* ^e13a3^ and pLEX-FLAG-*BCR-ABL1* ^e14a3^ we employed the Q5 site-direct mutagenesis kit according to the manufacturer’s protocol (NEB) using the following primers:

Fw^INS/Del^: 5′-ATA​TTC​AGA​GTT​TCA​TGT​TCT​GAA​GCC​GCT​CGT​TGG​AA-3′
Rv^INS/Del^: 5′-TTG​GCC​ATT​ATT​CTG​GCA​ATT​GTT​CTC​CCT​CCA​CTC​TGC-3′
Fw^e13a3^: 5′-TTC​CTT​ATT​GAT​GGT​CAG​CGG​A-3′
Rv^e13a3^: 5′-GGT​GAA​AAG​CTC​CGG​GTC​TTA​G-3′
Fw^e14a3^: 5′-AAG​TGA​AAA​GCT​CCG​GGT-3′
Rv^e14a3^: 5′-GAA​CTC​TGC​TTA​AAT​CCA​GTG-3′

Each FLAG-tagged *BCR-ABL1* deletion construct was used as a template to obtain the respective 6xHIS-tagged-derivative employing the primers indicated below:

Fw^6xHIS-BCR^: 5′-ACT​AGT​GCC​ACC​ATG​CAT​CAC​CAT​CAC​CAT​CAC​ATG​GTG​GAC​CCG​GTG​GGC-3'
Rv^ABL1^: 5′-ACG​CGT​CTA​CCT​CTG​CAC​TAT​GTC​ACT-3′

PCR products were ligated using the Spe-I and Mlu-I enzymes.

The pLEX-Myc-FLAG vector was generated amplifying human c-Myc by RT-PCR using total RNA extracted from HL-60 cells. The following primers were used:

Fw^Myc^: 5′-ACC​GGT​GCC​ACC​ATG​CCC​CTC​AAC​GTT​AGC​TTC​A-3'.
Rv^FLAG-Myc^: 5′-GCG​GCC​GCT​TAC​TTA​TCG​TCG​TCA​TCC​TTG​TAA​TCC​GCA​CAA​GAG​TTC​CGT​AGC​TG-3′.

The PCR product was then cloned in pLEX-EV using the Age-I and Not-I restriction sites.

### Lentivirus Production, Titering and Transduction

Recombinant lentiviral production, titration and transduction were performed as previously reported (Tirro et al., 2019a). Lentiviral particles were obtained according to the Dharmacon protocol as previously described (Jiang et al., 2015) and then titrated using the Lenti-X p24 Rapid Titer Kit (TakaraBio) according to the manufacturer’s protocol.

All cell lines were transduced by two rounds of spinoculation at 1,200 x g for 90 min at 32°C in the presence of 8 µg/ml of polybrene (Sigma) for Ba/F3 and Rat1 and 4 µg/ml of polybrene for CD34-positive cells. Primary CD34-positive progenitors were maintained in Stem Span SFEM supplemented with 100 ng/ml Flt-3 ligand, 100 ng/ml stem cell factor, 20 ng/ml interleukin-3 (IL-3) and interleukin-6 (IL-6). Rat1 cells were lentivirally transduced (MOI = 5) with pLEX-Myc-FLAG while Ba/F3 (MOI = 10) were exposed to the pLEX-EV and FLAG-tagged *BCR-ABL1* constructs reported above. All transduced cells received 3.5 µg/ml puromycin to select resistant clones. Rat1^Myc^ (MOI = 10) and CD34-positive cells (MOI = 70) were lentivirally infected with *BCR-ABL1* ^WT^ and deletion mutants and used for the indicated experiments.

### Transformation Assays

To assess BCR-ABL1-mediated growth factor-independent transformation, lentivirally transduced Ba/F3 cells were deprived of IL-3 for 24 h. At this time, 1 × 10^4^/ml were cultivated in the absence of IL-3 and counted every 24 h for three days. 1 × 10^4^/100 µL CD34-positive progenitors ectopically expressing native and atypical BCR-ABL1 fusion transcripts, were exposed to IM, NIL, DAS or PON and counted after three days. For both immortalized and primary cell populations, their number was obtained by trypan blue exclusion assays using a 0.4% trypan blue solution.

### Colony Forming Units Assay

Lentivirally infected CD34-positive cells were either grown in the absence of drugs or exposed to IM, NIL, DAS or PON for 24 h. At this time, 1 × 10^3^ cells were dispersed in TKI-additioned methylcellulose (Stem Cell Technologies) and 15 days later the resulting colonies were counted under an optical microscope (IX71; Olympus). Only colonies with >20–30 cells were considered (Aloisi et al., 2006).

### LTC-IC

Bone marrow leukemic CD34-positive cells obtained from CML patients at diagnosis and expressing the e14a3 fusion transcript, were exposed to IM, NIL, DAS or PON. After 24 h, cells were seeded on an M2-10B4 mouse fibroblasts feeder in 96 well-plates replacing the LTC-IC medium (MyeloCult H5100, Stem Cell Techonologies) weekly. After 5 weeks of culture, each well was overlaid with methylcellulose (H4435, Stem Cell Theconologies) supplemented with 10% of conditioned medium derived from 5,637 cells (Konig et al., 2008). Colonies were scored under an optical microscope after 12 additional days and the LTC-IC frequency calculated using the L-Calc software (Stem Cell Tecnologies).

### Protein Purification and *In Vitro* Kinase Assay

To purify 6xHIS-tagged BCR-ABL1^WT^, BCR-ABL1^∆DC2^, BCR-ABL1^∆SH3^, BCR-ABL1^INS/Del^, BCR-ABL1^e13a3^ and BCR-ABL1^e14a3^, each lentiviral vector was transiently transfected by calcium phosphate in HEK293 cells. Forty-eight hours post-transfection, a purification step was performed employing the Ni-NTA purification system (Thermo Fisher Scientific). *In vitro* kinase assays were performed as previously reported (Massimino et al., 2014) employing ADP-GLO (Promega), using approximately 8 nM of each BCR-ABL1 construct and concentrations of the ABLTIDE peptide [EAIYAAPFAKKK] (SignalChem) ranging from 0.78 to 100 µM. The resulting data was then used to calculate the ADP-ATP conversion rate thus obtaining the *Km* and *Vmax* values (analyses were performed with the Prism software). The constant catalytic rate (*Kcat*) was obtained from the equation *Kcat* = *Vmax*/[enzyme] and *Kcat*/*Km* ratio defining catalytic efficiency.

### Immunoblots

Whole cell lysates were prepared by resuspending Ba/F3 or Rat1 cells in Laemmli buffer. Cell pellets were then sonicated, denatured and protein lysates separated by SDS-PAGE. Antibodies used were as follow: anti-phosphotyrosine (4G10; Millipore, 2138006), anti-actin (AC-15, 121M4846) and anti-FLAG-M2-F3165 (both from Sigma, SLBN8915V), anti-MYC (9E10; Santa Cruz, (H1314), anti-AKT rabbit (9272, 28), anti-p44-42 mitogen-activated protein kinase rabbit (ERK1/2, 9102, 27), anti-STAT5 rabbit (D206Y- 9363, 1), anti-CRKL mouse (32H4, 3182, 5) and phospho-specific anti-bodies anti-pAKT-Ser473 rabbit (S473, 9271, 14), anti-p44-42 mitogen-activated protein kinase rabbit (ERK1/2, Thr202-Tyr204, 9101, 30), pSTAT5-Y694 rabbit (9351 9) and pCRKL-Y207 rabbit (3181,5), all from Cell Signaling. Appropriate horseradish peroxidase-conjugated secondary antibodies were used to detect the indicated proteins using the LiteAblot enhanced chemiluminescence reagent (EuroClone, MI, Italy) and signals were acquired using the C-DIGIT system (Licor).

### MTS and IC_50_ Calculation

Infected Ba/F3 cells were deprived of IL-3 for 24 h. At this time, 4 × 10^3^/100 µL cells in 96 well-plates were exposed to logarithmic dilutions of IM, NIL, DAS or PON. Three days later, the CellTiter 96^®^ AQueous One Solution Cell Proliferation Assay (Promega) was used to quantify cell viability according to the manufacturer’s protocol. The absorbance obtained was used to calculate IC_50_ values employing the Prism software (GraphPad Software).

### Growth Competition Assay Experiments

Ba/F3 cells expressing *BCR-ABL1* ^WT^ were individually mixed in a 1:1 ratio with those transduced with *BCR-ABL1* ^∆DC2^, *BCR-ABL1* ^∆SH3^, *BCR-ABL1* ^INS/Del^, *BCR-ABL1* ^e13a3^ and *BCR-ABL1* ^e14a3^ (Griswold et al., 2006). Each individual cell mixture was then exposed to IM, NIL, DAS or PON replacing fresh medium additioned with drugs every three days and re-implanting cells at the same confluence. Each cell mixture was then harvested and used for total RNA extraction as reported in Figure 3F.

### RNA Extraction and One-Step RT-PCR

For transduced Ba/F3 cells, total RNA was extracted using the Trizol reagent following the manufacturer’s protocol (Termofisher Scientific). For plucked CD34-derived CFUs, RNA was extracted as previously described (Tirro et al., 2019c).

The following primers were used to amplify the *BCR-ABL1* oncogene:

Fw^BCR10^: 5′-TAT​GAC​TGC​AAA​TGG​TAC​ATT​CC-3′
Rv^ABL4^: 5′-TCG TAG TTG GGG GAC ACA CC-3′.

Primers recognize exons 10 and 4 of *BCR* and *ABL1* (Massimino et al., 2019a; Massimino et al., 2019b).

To assess the integrity of the RNA derived from CD34-positive cells, CD45 was used and amplified employing the following primers:

Fw^CD45^: 5′-ACA​GCC​AGC​ACC​TTT​CCT​AC-3′
Rv^CD45^: 5′-GTG​CAG​GTA​AGG​CAG​CAG-3′.

### Anchorage-Independent Growth

Anchorage-independent assays were performed as previously reported (Lugo and Witte, 1989; Sahay et al., 2008; Sears et al., 2010) with the following modifications. 20 × 10^3^/cm^2^ transduced Rat1 cells were cultivated to achieve their confluence and subsequently over-grown for additional 10 days until foci formation was visible under an optical microscope. Cells were then trypsinized and re-implanted to repeat the over-growth process to obtain more than 80% cells showing foci formation. At this time, adherent and non-adherent cells were either left untreated or exposed to IM, NIL, DAS or PON. Two days later, 15 × 10^3^ cells for each condition were dispersed in soft-agar medium (D-MEM high glucose added of TKIs, 10% FBS, 2 mM glutamine and containing 0.3% agar) and stratified on a soft-agar bottom phase (soft-agar medium containing 0.6% agar). Each well was overlaid with growth medium to feed cells every 7 days. Twenty days later, each well was covered with an MTT (Sigma) solution (5 mg/ml in D-MEM high glucose) and placed in a CO_2_ incubator for 4 h. Images were acquired and colony numbers counted using the Image-J software.

### Statistical Analyses

The Prism Software was used to perform analysis of variance (One-way ANOVA).

## Results

### The *BCR-ABL1* Breakpoint Region Modulates the Oncoproteins Catalytic Efficiency

**FIGURE 1:**
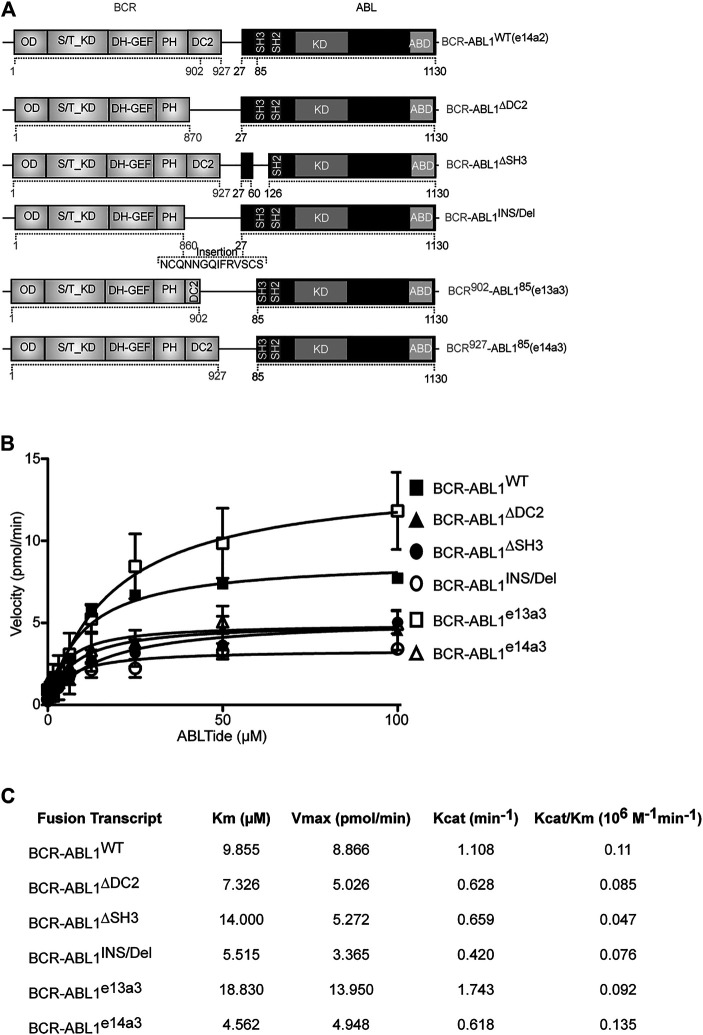
BCR-ABL1 catalytic efficiency is modulated by its breakpoint region. **(A)** Schematic representation of the BCR-ABL1 constructs used in this study. Numbers indicate the amino acid positions involved in the deleted region. **(B)** Curves show the results of an *in vitro* kinase assay plotting velocity versus substrate concentration using the indicated 6xHIS constructs. Assays were performed in triplicates with the standard deviation of the velocities shown as error bars. **(C)** Panel reporting the enzymatic values of substrate-binding rates expressed as the Michaelis-Menten constant (*Km*), maximum reaction rate (*Vmax*), the catalytic constant rate (*Kcat*) and the *Kcat*/*Km* ratio defining catalytic efficiency. OD: Oligomerization Domain; S/T_KD: Serine Threonine Kinase Domain; DH-GEF (Dbl Homology Domain-Guanine Nucleotide Exchange Factor); PH (Pleckstrin Homology Domain); DC2: C2 domain; SH3-SH2: Src homology domains; KD: kinase domain, ABD: Actin Binding Domain.

Previous evidence has demonstrated autoinhibition of ABL1 kinase activity by the SH3 domain (Smith et al., 2003; Hantschel, 2012). Furthermore, an inverse correlation has been reported between the size of the BCR portion retained in the oncogenic fusion and CML outcome (Yao et al., 2017). These findings suggest a direct involvement of the *BCR-ABL1* breakpoint region in modulating the oncoproteins catalytic activity. To assess the role of the DC2 and SH3 domains in regulating kinase efficiency, we generated five different *BCR-ABL1* fusion transcripts (Figure 1A) and employed an *in vitro* kinase assay to define their catalytic activity as compared to native BCR-ABL1. Using a preferential ABL1 substrate we analyzed the effects of varying substrate concentration on each enzymatic reaction. Applying the nonlinear regression fits, we calculated the Michaelis-Menten constant (*Km*), the maximum reaction rate (*Vmax*), the catalytic constant rate (*Kcat*) and the catalytic efficiency (*Kcat*/*Km*) of each mutant (Figures 1B,C). Compared to BCR-ABL1^WT^, we found that BCR-ABL1^∆SH3^ and BCR-ABL1^e13a3^ displayed a 1.4-fold and a 1.9-fold higher *Km* value, suggesting reduced substrate binding to the active site. On the contrary, we observed lower *Km* values (1.34, 1.79 and 2.2-fold) for BCR-ABL1^∆DC2^, BCR-ABL1^INS/Del^ and BCR-ABL1^e14a3^, indicating higher substrate affinity. Analyzing the maximum rate of the enzymatic reaction (*Vmax*), we found lower saturation concentrations (1.76, 1.70, 2.63 and 1.80-fold) for BCR-ABL1^∆DC2^, BCR-ABL1^∆SH3^, BCR-ABL1^INS/Del^ and BCR-ABL1^e14a3^. On the contrary, BCR-ABL1^e13a3^ presented a 1.57-fold higher *Vmax* than BCR-ABL1^WT^. To establish the catalytic rate of each atypical *BCR-ABL1* isoform we divided the detected *Vmax* for the enzyme concentration employed in the assay, thus calculating the *Kcat* value. We found that BCR-ABL1^e13a3^ showed a 1.57-fold higher *Kcat* value, while all the remaining constructs presented values lower than BCR-ABL1^WT^. Finally, to establish if the *BCR-ABL1* breakpoint region modifies the oncoprotein’s catalytic efficiency, we divided the *Kcat* of each isoform for its *Km* determining that BCR-ABL1^∆DC2^, BCR-ABL1^∆SH3^, BCR-ABL1^INS/Del^ and BCR-ABL1^e13a3^ were less catalytically efficient than BCR-ABL1^WT^. Unlike the aforementioned isoforms, BCR-ABL1^e14a3^ displayed a *Kcat*/*Km* ratio 1.23-fold higher than the native construct. These findings strongly suggest that the *BCR-ABL1* breakpoint region modulates the oncoproteins substrate binding, substrate affinity and kinase activity.

### Alterations of the *BCR-ABL1* Breakpoint Region Modify Its Transforming Potential and the Activation of Its Downstream Targets

**FIGURE 2:**
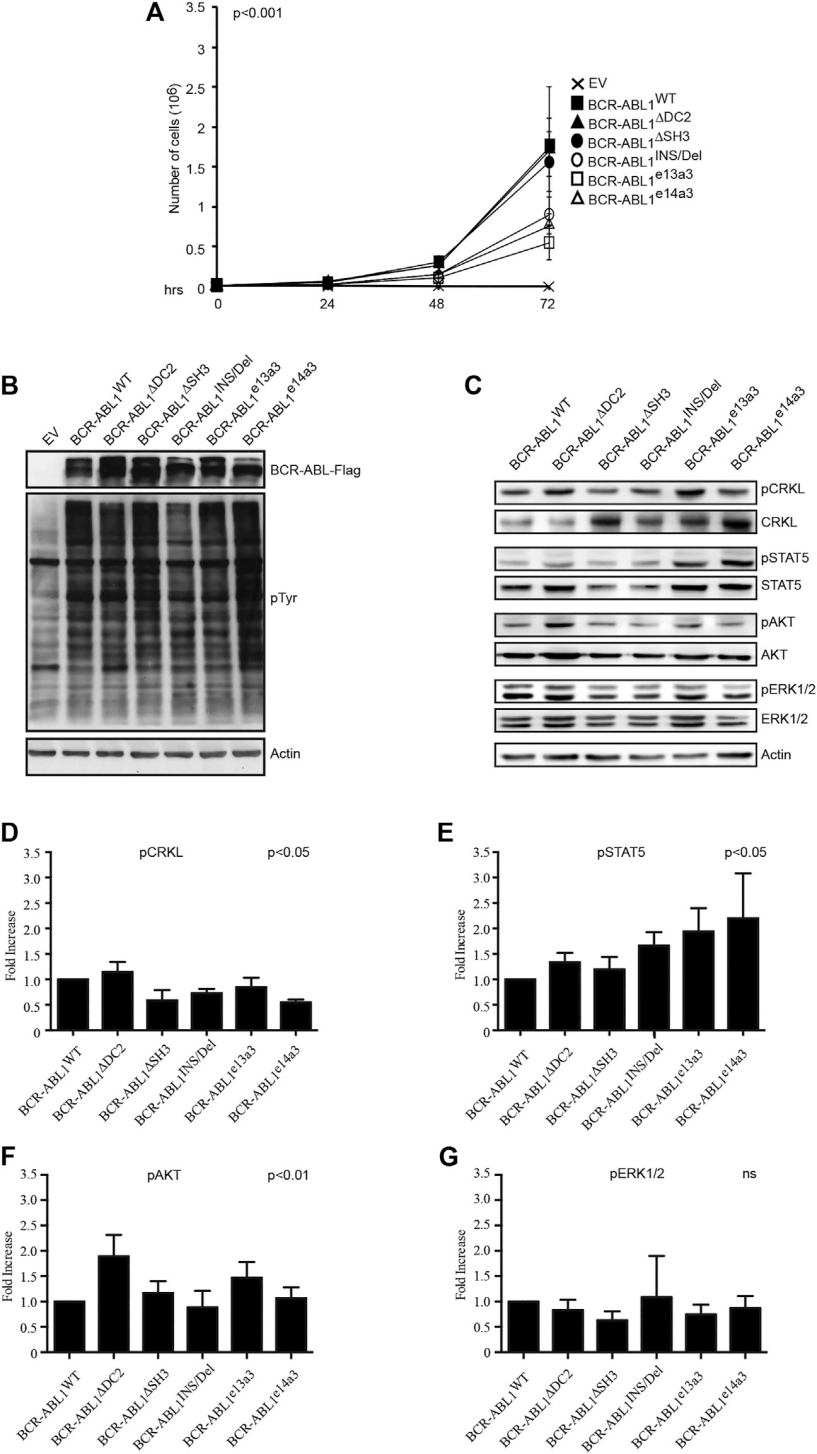
Alterations in the breakpoint region modify BCR-ABL1-dependent transforming ability and downstream target phosphorylation. **(A)** Curves indicate the number of viable Ba/F3 cells transduced with the specified constructs as determined by the Trypan blue exclusion assay. Bars indicate standard deviation derived from three independent experiments performed in triplicates. **(B**,**C)** Immunoblots of protein lysates obtained from transduced Ba/F3 cells. Protein lysates were separated by SDS-PAGE, transferred to nitrocellulose membranes and hybridized with anti-FLAG, anti-posphtyrosine **(B)** or other antibodies recognizing the indicated total or phosphorylated protein **(C)**. For all immunoblots actin was used as loading control. **(D**–**G)** Histograms reporting the densitometric analysis for each indicated phosphorylated protein derived from the immunoblot showed in **(B)** employing the Image J software and arbitrarily setting the densitometric value of BCR-ABL1^WT^ at 1. The densitometric value of each total and phospho-protein was initially normalized to actin. The final relative densitometric units were obtained by calculating the ratio between phosphorylated versus total protein fractions. Bars indicate standard deviations derived from two independent experiments.

It has been previously reported that different BCR-ABL1 fusion proteins lead to different disease phenotypes (Hur et al., 2002; Hanfstein et al., 2014; Jain et al., 2016). To better investigate the impact of alterations in the *BCR-ABL1* breakpoint region on cell transformation and intracellular signaling, we lentivirally expressed *BCR-ABL1* ^WT^ and five atypical fusion transcripts in Ba/F3 cells. We initially evaluated the ability of native and atypical transcripts to mediate IL3-independent growth. We observed that BCR-ABL1^WT^ was more proficient in inducing cytokine-independent growth than BCR-ABL1^INS/Del^, BCR-ABL1^e13a3^ and BCR-ABL1^e14a3^, while BCR-ABL1^∆DC2^ and BCR-ABL1^∆SH3^ showed comparable transforming activity (Figure 2A). We subsequently investigated if these differences were dependent on modifications of the intracellular tyrosine phosphorylation pattern. To this end, we first performed a total anti-phosphotyrosine immunoblot and found that—compared to the empty vector condition—all *BCR-ABL1* constructs induced higher phosphorylation levels. However, we did not observe visible differences in the immunoreactivity of Ba/F3 cells expressing BCR-ABL1^WT^ or the various deletion mutants. These results indicate that uncommon breakpoints do not majorly affect BCR-ABL1-dependent intracellular tyrosine phosphorylation (Figure 2B). We therefore investigated if atypical breakpoint regions can modulate BCR-ABL1-dependent phosphorylation of its direct (CRKL, STAT5) and indirect (AKT, ERK1/2) substrates (Figure 2C). Compared to native BCR-ABL1, densitometric analyses revealed that only BCR-ABL1^∆DC2^ and BCR-ABL1^e13a3^ preserved CRKL phosphorylation (pCRKL) while the remaining atypical transcripts showed a reduced ability to phosphorylate this protein (Figure 2D). Furthermore, although all BCR-ABL1 deletion mutants increased STAT5 phosphorylation, we observed that BCR-ABL1^e14a3^ and BCR-ABL1^e13a3^ were more effective than native BCR-ABL1 (Figure 2E). We subsequently investigated the activation of BCR-ABL1-indirect downstream targets and found that pAKT levels were increased in Ba/F3 cells expressing BCR-ABL1^∆DC2^ and BCR-ABL1^e13a3^ (Figure 2F) while no variations were observed for the remaining deletion mutants. No statistically significant differences were detected in ERK1/2 phosphorylation (Figure 2G). These data indicate that the BCR-ABL1 breakpoint region plays a critical role in modulating the activation of intracellular transducers involved in cell survival (STAT5) and anti-apoptotic signaling (AKT).

### Ba/F3 Cells Expressing Uncommon *BCR-ABL1* Transcripts are More Sensitive to Nilotinib

**FIGURE 3:**
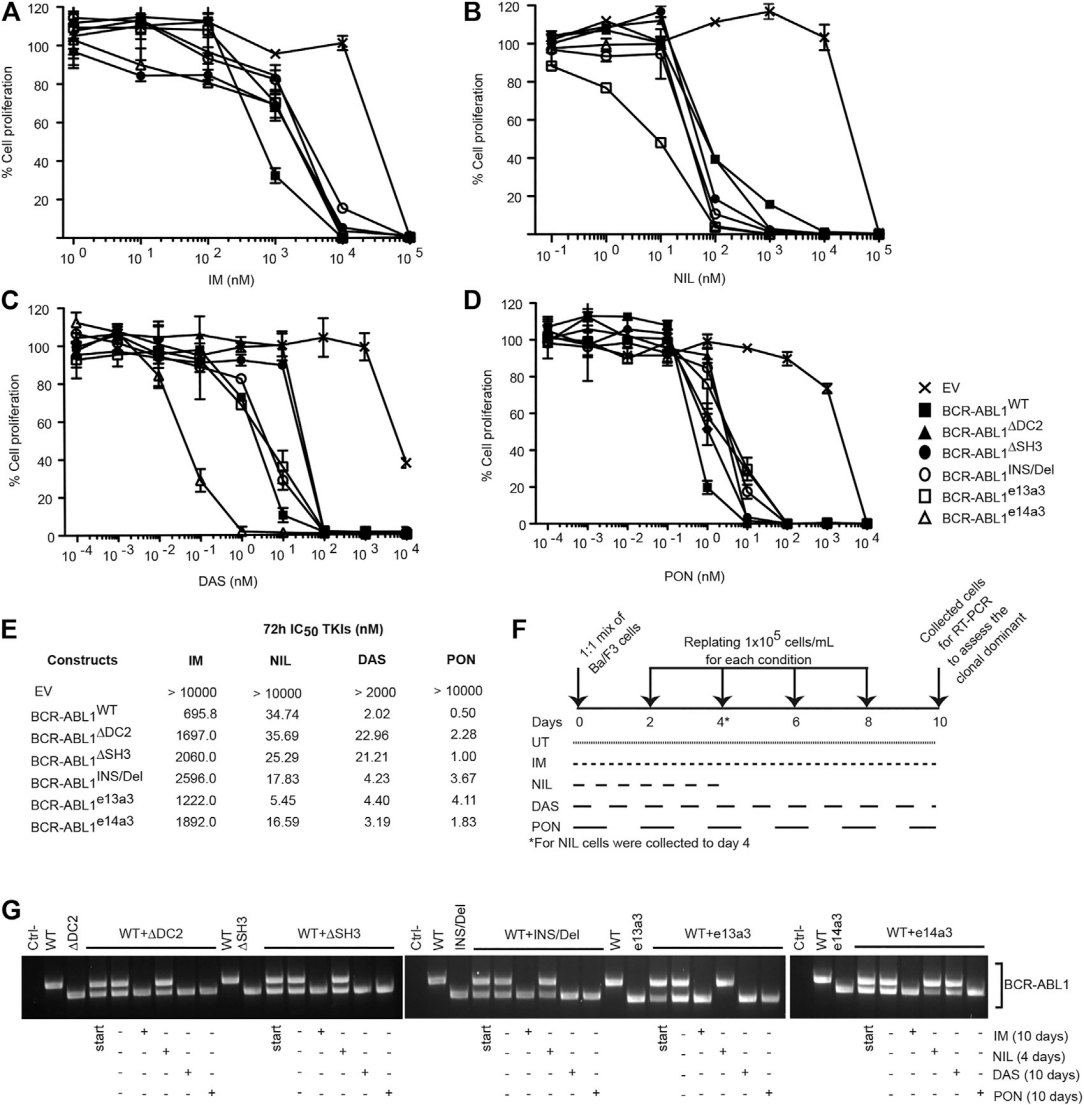
Ba/F3 cells transformed by atypical *BCR-ABL1* fusion transcripts are more sensitive to NIL than IM, DAS and PON. **(A**–**D)** MTS assay to define the IC_50_ values of Ba/F3 cells expressing the indicated *BCR-ABL1* constructs after exposure to logarithmic dilutions of the specified TKIs for 72 h. Bars indicate the standard deviations obtained from three independent experiments performed in triplicates. **(E)** IC_50_ values for each cell line were calculated using the Prism Software^®^. **(F)** Schematic representation of the TKI treatment in the growth competition experiment reported in **(G)**. **(G)** RT-PCR on total RNA extracted from Ba/F3 cell co-cultures collected after 10 days (IM, DAS and PON) or 4 days (NIL).

To establish if atypical breakpoints affect BCR-ABL1 responsiveness to different TKIs, we exposed Ba/F3 cells expressing BCR-ABL1^WT^ and the five deletion mutants to logarithmic concentrations of IM, NIL, DAS and PON and calculated their IC_50_ values (Figures 3A–D). We observed that cells expressing uncommon *BCR-ABL1* transcripts required higher concentrations of IM, DAS and PON to inhibit their proliferative activity. Strikingly, this was not the case after NIL exposure. In detail, after NIL treatment BCR-ABL1^WT^ and BCR-ABL1^∆DC2^ showed comparable IC_50_ values, while BCR-ABL1^∆SH3^, BCR-ABL1^INS/Del^ BCR-ABL1^e13a3^ and BCR-ABL1^e14a3^ displayed IC_50_ values that were 1.37, 1.95, 6.3 and 2.16-fold lower than the common oncoprotein (Figure 3E). Subsequently we wanted to determine the clonal growth advantage of Ba/F3 cells expressing BCR-ABL1 mutants comparing them to BA/F3 cells expressing native BCR-ABL1 after a long time TKIs exposure. To this end we used the native BCR-ABL1 IC_50_ values to establish if selectively reduces clonal growth when different deletion mutants were mixed with each other in a cell growth competition assay. We mixed an equal number of Ba/F3 cell expressing BCR-ABL1^WT^ with each of the uncommon fusion transcripts obtaining a series of co-cultures. Each co-culture was then individually exposed to the BCR-ABL1^WT^ IC_50_ value for each TKI, as reported in Figure 3F. We found that after 10 days of co-culture, Ba/F3 cells expressing BCR-ABL1^∆DC2^, BCR-ABL1^∆SH3^, BCR-ABL1^INS/Del^ and BCR-ABL1^e13a3^ successfully outgrew their BCR-ABL1^WT^ counterpart in the presence of IM, DAS and PON (Figure 3G). We obtained the same results for BCR-ABL1^e14a3^ with the exception of DAS, which showed comparable efficacy on this mutant and on BCR-ABL1^WT^. Interestingly, when we exposed each co-culture to NIL, we found that native BCR-ABL1 and the atypical transcripts BCR-ABL1^∆DC2^, BCR-ABL1^∆SH3^, BCR-ABL1^INS/Del^ and BCR-ABL1^e14a3^, were equally sensitive to this TKI. Furthermore, BCR-ABL1^e13a3^ was more susceptible to NIL than BCR-ABL1^WT^. These results imply that modifications in the *BCR-ABL1* breakpoint region are critical for the activity of most ABL1 inhibitors with the exception of NIL.

### The *BCR-ABL1* Breakpoint Region is Critical for the Anchorage-Independent Growth of Rat1^Myc^ Cells

**FIGURE 4:**
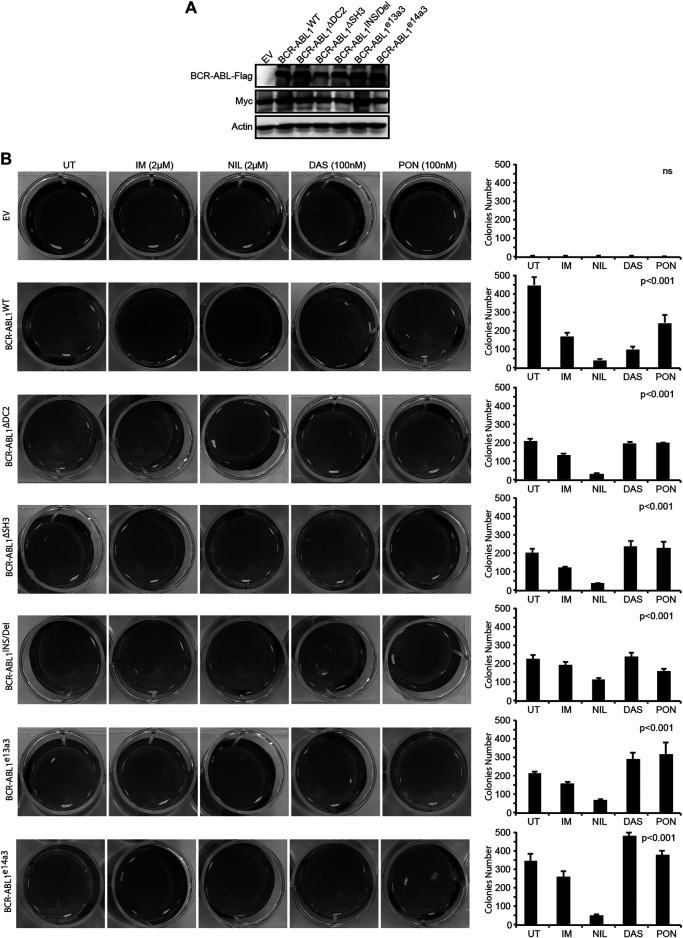
Uncommon breakpoint regions influence BCR-ABL1-dependent defective adhesion and show high sensitivity to NIL in Rat1 cells. **(A)** Immunoblots of Rat1 cells transduced with the indicated constructs. Protein lysates were obtained and processed as detailed in Figure 2. Actin was used as loading control. **(B)** Image showing colony formation in soft-agar of Rat1 cells expressing the indicated constructs. Bars report the standard deviations obtained from two independent experiments.

Jongen-Lavrencic and others have previously shown that BCR-ABL1 induces defective cell adhesion and impaired migration (Jongen-Lavrencic et al., 2005). These findings are in keeping with available evidence indicating that the oncoprotein induces fibroblasts transformation by promoting anchorage-independent growth (Smith et al., 2003). To establish if the BCR-ABL1 breakpoint region modulates this effect, we lentivirally expressed BCR-ABL1^WT^ and the five atypical fusion transcripts in Rat1^Myc^ cells. These cells represent a helpful tool to investigate BCR-ABL1-dependent adhesion since, when overexpressing the human *cMyc* gene, they can be transformed by BCR-ABL1 (Lugo and Witte, 1989; Sahay et al., 2008; Gong et al., 2017). After confirming the correct expression of both MYC and BCR-ABL1 (Figure 4A), Rat1 transduced cells were exposed to the equivalent plasma concentration of each TKI (Bradeen et al., 2006) and employed to perform an anchorage-independent assay (Figure 4B). As expected, Rat1^Myc-EV^ cells were unable to grow in the absence of adhesion while Rat1^Myc^ expressing BCR-ABL1^WT^ or the five deletion mutants acquired this ability. Specifically, BCR-ABL1^WT^ was more proficient in promoting anchorage-independent growth than BCR-ABL1^∆DC2^, BCR-ABL1^∆SH3^, BCR-ABL1^INS/Del^, BCR-ABL1^e13a3^ and BCR-ABL1^e14a3^. Interestingly, all TKIs reduced the colony-forming ability of both native BCR-ABL1 and the five tested constructs. However, DAS and PON failed to inhibit anchorage-independent growth mediated by uncommon *BCR-ABL1* transcripts. These observations implicate the BCR-ABL1 breakpoint region in the modulation of cell adhesion.

### The *BCR-ABL1* Breakpoint Region Contributes to the Transforming and Clonogenic Properties of the Oncoprotein in Human CD34-Positive Progenitors

**FIGURE 5:**
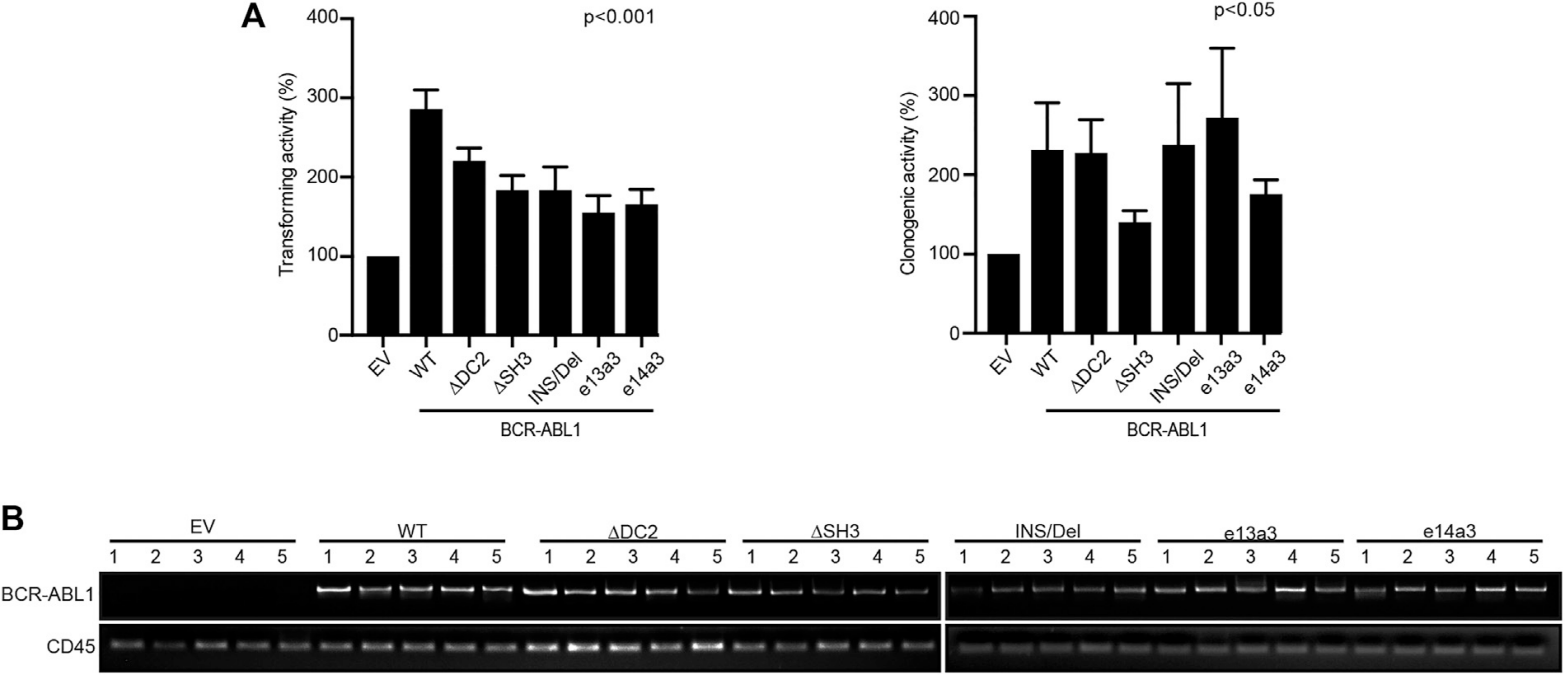
Uncommon fusion transcripts modify BCR-ABL1 transforming ability and clonogenic potential. **(A)** Histograms show the percentage of transforming ability (I) and clonogenic potential (II) of the indicated constructs lentivirally expressed in human CD34-positive cells compared to the empty vector condition arbitrarily set at 100%. Bars indicate the standard deviation of two experiments performed in duplicates. **(B)** RT-PCR performed on total RNA extracted from five single colonies plucked after 15 days of growth in methylcellulose. CD45 was used as an RNA integrity control.

To investigate if the *BCR-ABL1* breakpoint region influences the transformation of CD34-positive cells, we lentivirally expressed BCR-ABL1^WT^ and the five uncommon fusion transcripts in human CD34-positive progenitors and evaluated both BCR-ABL1-dependent transformation and cell clonogenicity. For transformation assays, cells were cultivated in presence of low cytokines concentrations to avoid impairing BCR-ABL1-dependent growth (Modi et al., 2007). We found that all *BCR-ABL1* constructs maintained their transforming ability. However, BCR-ABL1^WT^ was more effective than any deletion mutant in transforming CD34-positive cells (Figure 5Ai). We then investigated if modifications in the breakpoint region can alter BCR-ABL1-mediated clonogenicity. We observed that all *BCR-ABL1* constructs increased colony numbers compared to the vector control but CD34 cells expressing BCR-ABL1^∆SH3^ and BCR-ABL1^e14a3^ were less clonogenic (Figure 5Aii). Successful lentiviral infection was confirmed by performing RT-PCR on five individual colonies selected from each condition (Figure 5B) (Aloisi et al., 2006). These results suggest that the *BCR-ABL1* breakpoint region is also implicated in the oncoproteins ability to transform CD34-positive hematopoietic progenitors and modulates its clonogenic potential.

### Nilotinib Inhibits Both the Transforming Potential and Clonogenic Ability of Atypical *BCR-ABL1* Transcripts in CD34-Positive Cells

We finally wanted to investigate the impact of different *BCR-ABL1* breakpoints on the oncoproteins TKI responsiveness. To this end, we exposed lentivirally transduced CD34-positive cells to IM, NIL, DAS and PON using doses corresponding to their achievable plasmatic concentration (Bradeen et al., 2006).

**FIGURE 6:**
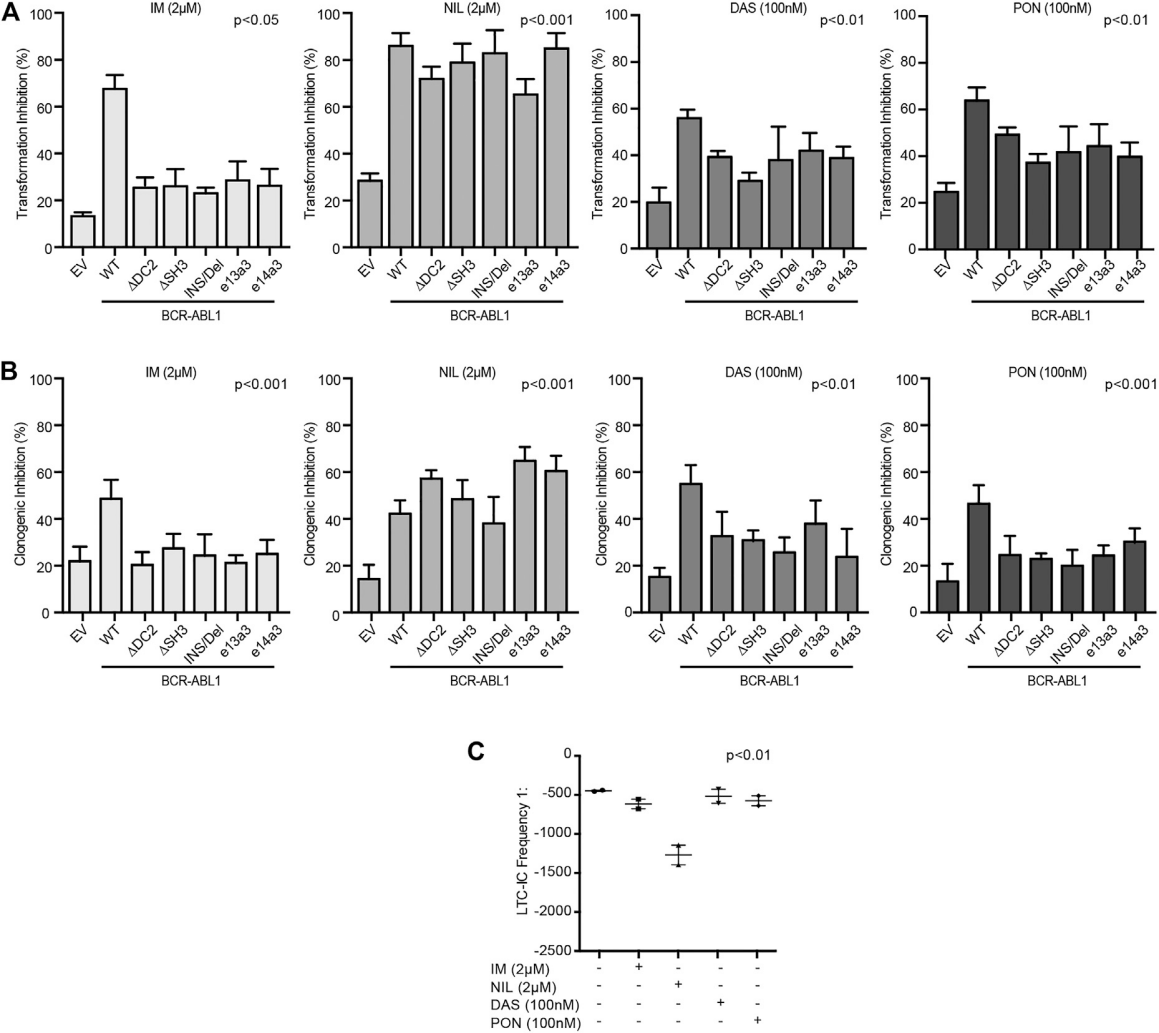
CD34-positive cells expressing atypical *BCR-ABL1* transcripts are highly sensitive to NIL. **(A**,**B)** Histograms indicating the percentage of transformation **(A)** and clonogenic **(B)** inhibition of CD34-positive cells expressing the indicated BCR-ABL1 constructs and exposed to different TKIs. Percentage values were obtained setting untreated cells for each condition to 100%. Bars indicate the standard deviation of two experiments performed in duplicates. **(C)** Dot plot showing the LTC-IC frequency of CD34-positive cells derived from a CML patient expressing e14a3 *BCR-ABL1* after no treatment or exposure to TKIs at the indicated concentrations. All experiments were performed in duplicates.

We observed that, while all TKIs inhibited the BCR-ABL1-mediated cell transformation and clonogenicity, NIL was more potent than IM, DAS and PON (Figures 6A,B). To confirm these results, we performed LTC-IC assays by LDA analysis on CD34-positive cells derived from a CML patient expressing the e14a3 variant. We confirmed that although IM, DAS and PON all reduced LTC-IC frequency, NIL was the more effective drug on these cells (Figure 6C). These findings support our previous results indicating NIL as the most potent TKI for the uncommon *BCR-ABL1* fusions included in our experimental models.

## Discussion

In the current study we investigated the impact of five different breakpoint regions on BCR-ABL1 leukemogenic potential and TKI responsiveness. Three of the five breakpoints were previously identified in CML patients (*BCR-ABL1* ^INS/Del^, *BCR-ABL1* ^e13a3^ and *BCR-ABL1* ^e14a3^) while two were purposefully engineered in our laboratory (*BCR-ABL1* ^∆DC2^ and *BCR-ABL1* ^∆SH3^). We found that these modifications affected BCR-ABL1 catalytic efficiency, signal transduction, transforming activity and reduced response to IM, DAS and PON but not NIL. Our work follows that of several other groups that have investigated the specific biological contributions of different functional domains within the BCR and ABL1 portions of the BCR-ABL1 chimeric oncoprotein. Nieborowska-Skorska and others have shown that a BCR-ABL1 construct devoid of either the SH2 or the SH3 domains retained the ability to activate STAT5, while STAT5 phosphorylation was lost after the simultaneous deletion of both domains (Nieborowska-Skorska et al., 1999). Accordingly, we found that total ablation of the SH3 domain (*BCR-ABL1* ^∆SH3^) did not affect STAT5 phosphorylation, while partial removal of the domain (as in *BCR-ABL1* ^e13a3^ and *BCR-ABL1* ^e14a3^) resulted in increased levels of phosphorylated STAT5. This unexpected finding suggests that alterations in the SH3-SH2 structure affect BCR-ABL1 substrate affinity and catalytic activity. Furthermore, as STAT5 phosphorylation was comparable between native BCR-ABL1 and mutants carrying deletions in BCR domains (*BCR-ABL1* ^∆DC2^ and *BCR-ABL1* ^INS/Del^), our results indicate that—within the breakpoint region—only ABL1 functional domains are required for STAT5 activation. On the contrary, BCR domains seem critical inhibitors of AKT activation as AKT phosphorylation increased in cells transduced with *BCR-ABL1* constructs displaying a reduced BCR contribution (*BCR-ABL1* ^∆DC2^ and *BCR-ABL1* ^e13a3^), supporting an inverse correlation between BCR size and activation of anti-apoptotic signaling. It should also be noted that, for the first time, our findings implicate the BCR-ABL1 breakpoint region in the regulation of cell adhesion as all five mutants included in the study were less proficient than native BCR-ABL1 in promoting anchorage-independent growth of Rat1^Myc^ cells.

Griswold and others have previously reported that mutations in the BCR-ABL1 kinase domain modify its substrate utilization and kinase activity (Griswold et al., 2006). These enzymatic characteristics were modified in all the mutants tested in this study, indicating that the functional domains encompassed in the BCR-ABL1 breakpoint region modulate the oncoproteins catalytic activity. Our results also demonstrate that, with the exception of the ABL1 SH3 domain, the remaining regions included in the *BCR-ABL1* breakpoint all contribute to the cytokine-independent transformation of Ba/F3 cells. Furthermore, while we confirm that STAT5 phosphorylation is required for BCR-ABL1-mediated growth factor independence (Maru et al., 1996; Skorski et al., 1998), we find that this event *per se* is insufficient to transform cytokine-dependent cells, as Ba/F3 expressing *BCR-ABL1* ^e13a3^ and *BCR-ABL1* ^e14a3^ showed high STAT5 phosphorylation while displaying reduced IL-3 independence. Interestingly, our results reiterate that BCR-ABL1 transforming potential and clonogenic ability are biologically and functionally distinct as the latter were mostly unaffected in the uncommon transcripts investigated in our experiments with the exception of *BCR-ABL1* ^∆SH3^.

Finally, we wanted to define the TKI responsiveness of the atypical *BCR-ABL1* transcripts included in this work. The introduction of ABL1-directed TKIs has dramatically improved the hematological, cytogenetic and molecular responses of leukemia patients displaying a *BCR-ABL1* chimeric fusion (Hochhaus et al., 2017; Ottmann et al., 2018; Saglio et al., 2018). Despite these excellent results, an ever-growing number of patients will either fail to achieve or loose a previously attained optimal response. TKI failure is driven by both BCR-ABL1-dependent and -independent mechanisms (Jordanides et al., 2006; White et al., 2007; Wu et al., 2008; Stagno et al., 2012; Castagnetti et al., 2017; Vigneri et al., 2017; Stella et al., 2019c; Soverini et al., 2020) often requiring additional therapeutic strategies (Buffa et al., 2014; Massimino et al., 2018). Published evidence proposes an inverse correlation between the size of the BCR contribution in the chimeric oncogene and TKI response (Jain et al., 2016; Short et al., 2016; Yao et al., 2017). Moreover, it has been shown that the ABL1 SH3 and SH2 domains exercise an important role in regulating the oncoprotein kinase activity (Filippakopoulos et al., 2009; Sherbenou et al., 2010; Grebien et al., 2011). These results suggest that modifications in the BCR and ABL1 domains encompassed in the BCR-ABL1 breakpoint may affect TKI response. Our data indicate that NIL was the most effective TKI in reducing the leukemogenic potential of all tested BCR-ABL1 mutants. These data are in agreement with our previous results where we demonstrated that CML patients with different *BCR-ABL1* variants were successfully treated with NIL (Massimino et al., 2019a; Tirro et al., 2019b; Manzella et al., 2020) but failed to respond to DAS (Massimino et al., 2019b). Accordingly, CML patients expressing *BCR-ABL1* ^INS/Del^ failed to obtain a good molecular response when exposed to IM or DAS but benefited from NIL treatment (Stella et al., 2019a). While the reasons underlying NIL superior efficacy remain unclear, we hypothesize that the drug’s prolonged intracellular accumulation and residence time when bound to the BCR-ABL1 kinase domain may contribute to the observed effect (Manley et al., 2010; Wagner et al., 2013). It should also be noted that, unlike DAS, IM and NIL fit in the ATP-binding site when a hydrophobic portion defined as the adenine binding-site region displays the Aspartate-Phenylalanine-Glycine motif (DFG) is in an out conformation (DFG-out, ABL kinase inactive conformation). However, despite this similarity, the different molecular structure of each compound explains their distinct structure-activity relationship. Indeed, IM requires stringent interactions by Van der Waals (VdW) forces and six highly energetic hydrogen bonds (H-bs) in order to dock to the adenine region, while NIL needs an increased number of VdW forces with a lower energetic contribution by H-bs (Rossari et al., 2018). Interestingly, although PON binds the adenine region in an IM/NIL-like mode (i.e., with the DFG-out), its interaction with the ABL1 kinase domain requires different amino acidic residues. These structural differences may explain why PON can overcome most mutation-dependent TKI resistance (Reddy and Aggarwal, 2012; Buffa et al., 2014).

Overall, these observations promote the hypothesis that the breakpoint region influences the structural conformation of the adenine region rendering NIL more effective than IM, DAS and PON against atypical *BCR-ABL1* fusion transcripts.

In summary, our findings demonstrate that the *BCR-ABL1* breakpoint region critically regulates the oncoproteins catalytic activity, transforming efficiency and TKI sensitivity and that the variations investigated in this work can reduce IM, DAS and PON sensitivity but not NIL responsiveness. Taken together our results suggest that CML patients expressing uncommon *BCR-ABL1* fusions may derive greater clinical benefit from NIL treatment.

## Data Availability

The raw data supporting the conclusions of this article will be made available by the authors, without undue reservation.
